# Predictive accuracy of enhanced versions of the on-admission National Early Warning Score in estimating the risk of COVID-19 for unplanned admission to hospital: a retrospective development and validation study

**DOI:** 10.1186/s12913-021-06951-x

**Published:** 2021-09-13

**Authors:** Muhammad Faisal, Mohammed Amin Mohammed, Donald Richardson, Ewout W. Steyerberg, Massimo Fiori, Kevin Beatson

**Affiliations:** 1 Faculty of Health Studies, University of Bradford, Bradford, UK; 2 Bradford Institute for Health Research , Bradford, UK; 3 NIHR Yorkshire and Humber Patient Safety Translational Research Centre (YHPSTRC), Bradford, UK; 4 Wolfson Centre for Applied Health Research, Bradford, UK; 5 The Strategy Unit, NHS Midlands and Lancashire Commissioning Support Unit, Kingston House, B70 9LD West Bromwich, UK; 6 Department of Renal Medicine, York Teaching Hospitals NHS Foundation Trust, York, England, UK; 7 Department of Public Health, Erasmus University, Rotterdam, The Netherlands; 8 Leiden University Medical Center, Leiden, The Netherlands; 9 York Teaching Hospitals NHS Foundation Trust, York, England, UK

**Keywords:** Vital signs, National early warning score, Emergency admission, Novel coronavirus SARS-19, Computer-aided national early warning score

## Abstract

**Background:**

The novel coronavirus SARS-19 produces ‘COVID-19’ in patients with symptoms. COVID-19 patients admitted to the hospital require early assessment and care including isolation. The National Early Warning Score (NEWS) and its updated version NEWS2 is a simple physiological scoring system used in hospitals, which may be useful in the early identification of COVID-19 patients. We investigate the performance of multiple enhanced NEWS2 models in predicting the risk of COVID-19.

**Methods:**

Our cohort included unplanned adult medical admissions discharged over 3 months (11 March 2020 to 13 June 2020 ) from two hospitals (YH for model development; SH for external model validation). We used logistic regression to build multiple prediction models for the risk of COVID-19 using the first electronically recorded NEWS2 within ± 24 hours of admission. Model M0’ included NEWS2; model M1’ included NEWS2 + age + sex, and model M2’ extends model M1’ with subcomponents of NEWS2 (including diastolic blood pressure + oxygen flow rate + oxygen scale). Model performance was evaluated according to discrimination (c statistic), calibration (graphically), and clinical usefulness at NEWS2 ≥ 5.

**Results:**

The prevalence of COVID-19 was higher in SH (11.0 %=277/2520) than YH (8.7 %=343/3924) with a higher first NEWS2 scores ( SH 3.2 vs YH 2.8) but similar in-hospital mortality (SH 8.4 % vs YH 8.2 %). The c-statistics for predicting the risk of COVID-19 for models M0’,M1’,M2’ in the development dataset were: M0’: 0.71 (95 %CI 0.68–0.74); M1’: 0.67 (95 %CI 0.64–0.70) and M2’: 0.78 (95 %CI 0.75–0.80)). For the validation datasets the c-statistics were: M0’ 0.65 (95 %CI 0.61–0.68); M1’: 0.67 (95 %CI 0.64–0.70) and M2’: 0.72 (95 %CI 0.69–0.75) ). The calibration slope was similar across all models but Model M2’ had the highest sensitivity (M0’ 44 % (95 %CI 38-50 %); M1’ 53 % (95 %CI 47-59 %) and M2’: 57 % (95 %CI 51-63 %)) and specificity (M0’ 75 % (95 %CI 73-77 %); M1’ 72 % (95 %CI 70-74 %) and M2’: 76 % (95 %CI 74-78 %)) for the validation dataset at NEWS2 ≥ 5.

**Conclusions:**

Model M2’ appears to be reasonably accurate for predicting the risk of COVID-19. It may be clinically useful as an early warning system at the time of admission especially to triage large numbers of unplanned hospital admissions.

**Supplementary Information:**

The online version contains supplementary material available at 10.1186/s12913-021-06951-x.

## Introduction

The novel coronavirus SARS-19 produces the newly identified disease ‘COVID-19’ in patients with symptoms (Coronaviridae Study Group of the International Committee on Taxonomy of Viruses [1]) which was declared as a pandemic on 11-March-2020 that has challenged healthcare systems worldwide.

COVID-19 patients admitted to hospital can develop severe disease with life-threatening respiratory and/or multi-organ failure [2, 3] with a high risk of mortality in part due to the lack of effective treatment for the underlying disease in the early phase of the pandemic. The appropriate early assessment and management of patients with COVID-19 is important in ensuring high-quality care including isolation, escalation to critical care or palliative care. Early assessment of the risk of COVID-19 is crucial to this process. Presently this involves clinical judgment based on the patients presenting history, signs and symptoms and viral nucleic acid testing can have a 24-hour turnaround time [4].

In England, the patient’s vital signs are monitored and summarised into a National Early Warning Score (NEWS) [5]. NEWS has gained widespread interest from across the world, including Europe, India, the USA (and the US Navy) [6]. NEWS offers a standardised approach to assessing acute illness and is derived from seven physiological variables or vital signs – respiration rate, oxygen saturations, any supplemental oxygen, temperature, systolic blood pressure, heart rate and level of consciousness (Alert (A), Voice (V), Pain (P), Unresponsive (U)) – which are routinely collected by nursing staff as an integral part of the process of care.

In December 2017, an update to NEWS (NEWS2) was published [6] that extends the level of consciousness from AVPU to ACVPU, where C represents new confusion or delirium and is allocated 3 points (the maximum for a single variable). NEWS2 also offers two scales for oxygen saturation (scale 1 and scale 2) which accommodates patients with hypercapnic respiratory failure who have clinically recommended oxygen saturation of 88–92 %.

We posit that NEWS and NEWS2, and their subcomponents, may be useful in predicting COVID-19 risk. So we investigate the performance of multiple enhanced NEWS and NEWS2 models in terms of discrimination and calibration in predicting the risk of COVID-19. We are using the first electronically recorded NEWS and NEWS2 datasets and are available within 24 h of admission. This means that the models we investigate require no additional data collection from staff and can be readily automated in electronic health records.

## Methods

### Setting and data

Our cohorts of unplanned medical admissions are from two acute hospitals which are approximately 65 km apart in the Yorkshire and the Humber region of England – Scarborough hospital (SH) (n ~ 300 beds) and York Hospital (YH) (n ~ 700 beds), managed by York Teaching Hospitals NHS Foundation Trust. We selected these hospitals because they had electronically recorded NEWS2 scores, which are collected as an integral part of the patient’s process of care and were agreeable to the study. Since NEWS is a subset of NEWS2, we developed NEWS and NEWS2 based models because NEWS is still in widespread use.

We included all consecutive adult (age ≥ 18 years) unplanned medical admissions discharged during 3 months (11 March 2020 to 13 June 2020), with electronic NEWS2 data. For each admission, we obtained a pseudonymised patient identifier, patient’s age (years), sex (male/female), discharge status (alive/dead), admission and discharge date and time, diagnoses codes based on the 10th revision of the International Statistical Classification of Diseases (ICD-10), NEWS2 (including its subcomponents respiratory rate, temperature, systolic pressure, pulse rate, oxygen saturation, oxygen supplementation, oxygen scales 2 (yes/no), and alertness including confusion). The diastolic blood pressure was recorded at the same time as systolic blood pressure. Historically, diastolic blood pressure has always been a routinely collected physiological variable on vital sign charts and is still collected where electronic observations are in place. Since NEWS is a subset of NEWS2, we derived NEWS from NEWS2. NEWS and NEWS2 produce integer values that range from 0 (indicating the lowest severity of illness) to 20 (the maximum NEWS2 value possible) (see Supplemental Digital Content - Table S1 and S2). The index NEWS/NEWS2 was defined as the first electronically recorded NEWS/NEWS2 within ± 24 h of the admission time. We excluded records where the index NEWS/NEWS2 was not within ± 24 h or was missing/not recorded at all (see Supplemental Digital Content - Table S3). We searched primary and secondary ICD-10 codes for ‘U071’ for identifying COVID-19. Although we used the ICD-10 code ‘*U071*’ to identify records with COVID-19, it is in 95 % agreement with polymerase chain reaction (PCR) swab tests result.

### Statistical analyses

We began with exploratory analyses including box plots that showed the relationship between covariates and risk of COVID-19 and line plots showed the relationship between age, vital signs, NEWS2 and risk of COVID-19. We developed three logistic regression models based on NEWS and NEWS2 separately for predicting the risk of COVID-19. The NEWS2-based models (M0’, M1’, M2’) use the index or first electronically recorded NEWS2 dataset within ± 24 h of admission. Model M0’ uses NEWS2 alone; Model M1’ extends M0’ with age and sex and Model M2’ extends M1’ with all the subcomponents of NEWS2 plus diastolic blood pressure. Equivalent models (M0, M1, M2) using NEWS were also developed but model M2 excluded two parameters that are in NEWS2 but no in NEWS - oxygen flow rate and scale 2 (yes/no). A log-transformation was used for variables with right-skewed distributions, i.e. for respiratory rate, pulse rate, systolic and diastolic blood pressure.

We developed all models using YH data (as development dataset) and externally validated their performance on SH data (as validation dataset).

We report discrimination and calibration statistics as performance measures for these models [7].

Discrimination relates to how well a model can separate, (or discriminate) between patients with and without COVID-19 and is given by the area under the Receiver Operating Characteristics (ROC) curve (AUC) or c-statistic. The ROC curve is a plot of the sensitivity, (true positive rate), versus 1-specificity, (false positive rate), for consecutive predicted risks. A c-statistic of 0.5 is no better than tossing a coin, whilst a perfect model has a c-statistic of 1. In general, values less than 0.7 are considered to show poor discrimination, values of 0.7 to 0.8 can be described as reasonable, and values above 0.8 suggest good discrimination [8]. The 95 % confidence interval for the c-statistic was derived using DeLong’s method as implemented in the *pROC* library [9] in R [10]. Calibration is the relationship between the observed and predicted risk of COVID-19 (24) and can be readily seen on a scatter plot (y-axis observed risk, x-axis predicted risk). Perfect predictions should be on the 45° line.

The predictive model performance is usually overestimated if the same data is used for testing model performance. There are several internal validation methods, which aimed to provide a more accurate estimate of predictive model performance. We used bootstrapping as an internal validation approach to assess the discrimination and calibration for all the models [11, 12]. The overall statistical performance was assessed using the scaled Brier score which simultaneously incorporates discrimination and calibration [7]. The Brier score is the squared difference between actual outcomes and predicted risk of COVID-19, scaled by the maximum Brier score such that the scaled Brier score ranges from 0 to 100 %. Higher Brier scores indicate superior models. We further assess discrimination and calibration-in-the-large and calibration slopes in the validation data.

The clinical cut-off of NEWS and NEWS2 is 5+ (Supplemental Digital Content - Figure S1). This is the recommended threshold for detecting deteriorating patients and sepsis [13, 14]. Therefore, we assessed the sensitivity, specificity, positive and negative predictive values and likelihood ratios for these models at NEWS/NEWS2 thresholds of 5+ [15]. We further compared the net benefit for all models, which may inform the utility of the models in routine clinical practice [16]. The net benefit is calculated at a particular threshold probability ${p}_{t}$ with total sample size $N$ as follows:

$$Net benefit= \frac{True positives}{N}-\frac{False positivies}{N}\times \frac{{p}_{t}}{1-{p}_{t}}$$

The model with the highest net benefit metric has the highest clinical value.

We calculated the minimum sample size using the R package *pmsampsize* [17]. We found 930 (93 events) is the minimum required sample size with number of predictors = 21, R2 = 0.182, prevalence = 0.10, shrinkage > 0.9, margin absolute prediction error (MAPE) = 0.05 [18]. We followed the TRIPOD guidelines for reporting model development and validation [19]. We have deployed our best performing models - M2’ and M2 - as a calculator for predicting the risk of COVID-19 https://covidcalc.shinyapps.io/calc/. We used Stata [20] for data cleaning and *R* [10] for statistical analysis.

## Results

### Cohort characteristics

The number of unplanned discharges was 6444 over 3 months. We excluded 36 (0.6 %) records because the index NEWS2 was not recorded within ± 24 h of the admission time or no recorded at all (see Supplemental Digital Content - Table S3).

The characteristics of the admissions included in our study are shown in Table 1. Emergency admissions in the validation dataset were older than those in the development dataset (69.6 years vs. 67.4 years), less likely to be male (49.5 % vs. 51.2 %), had higher index NEWS (2.8 vs. 2.5) and NEWS2 (3.2 vs. 2.8) scores, higher prevalence of COVID-19 (11.0 % vs. 8.7 %) but similar in-hospital mortality (8.4 % vs. 8.2 %). See accompanying scatter and boxplots in Supplemental Digital Content - Figure S2, S3, S4 and S5.

**Table 1:** Characteristics of emergency medical admissions discharged during 3 months in development and validation datasets from YH and SH hospitals

Characteristic	Development dataset (YH)	Validation dataset (SH)
N	3924	2520
Male (%)	2010 (51.2)	1247 (49.5)
Mean Age [years] (SD)	67.4 (18.7)	69.6 (18.9)
Median Length of Stay (days) (IQR)	3.0 (5.8)	3.7 (6.1)
COVID-19 (%)	343 (8.7)	277 (11.0)
**Mortality**		
Mortality with-in 24 h (%)	30 (0.8)	32 (1.3)
Mortality with-in 48 h (%)	61 (1.6)	48 (1.9)
Mortality with-in 72 h (%)	96 (2.4)	68 (2.7)
In-hospital Mortality	323 (8.2)	212 (8.4)
Mean NEWS (SD)	2.5 (2.3)	2.8 (2.4)
Mean NEWS2 (SD)	2.8 (2.8)	3.2 (2.8)
**Vital Signs**		
Mean Respiratory rate [breaths per minute] (SD)	19.8 (5.1)	20.7 (5.6)
Mean Temperature [^o^C] (SD)	36.4 (0.9)	36.3 (1)
Mean Systolic pressure [mmHg] (SD)	141.8 (29.2)	142 (28.5)
Mean Diastolic pressure [mmHg] (SD)	79.2 (16.5)	79 (17.3)
Mean Pulse rate [beats per minute] (SD)	89.1 (22.3)	88.5 (22.1)
Mean Oxygen saturation (SD)	96.3 (3.1)	96.1 (3.2)
Oxygen supplementation (%)	512 (13)	362 (14.4)
Mean oxygen flow rate (units) (SD)	7.1 (5.7)	6.1 (5.3)
Oxygen scale 2 (yes) (%)	240 (6.1)	163 (6.5)
**Alertness**		
Alert (%)	3510 (89.4)	2243 (89)
Baseline confusion (%)	27 (0.7)	23 (0.9)
New confusion (%)	61 (1.6)	40 (1.6)
Pain (%)	32 (0.8)	17 (0.7)
Voice (%)	151 (3.8)	134 (5.3)
Unconscious (%)	143 (3.6)	63 (2.5)

**Fig. 1:**
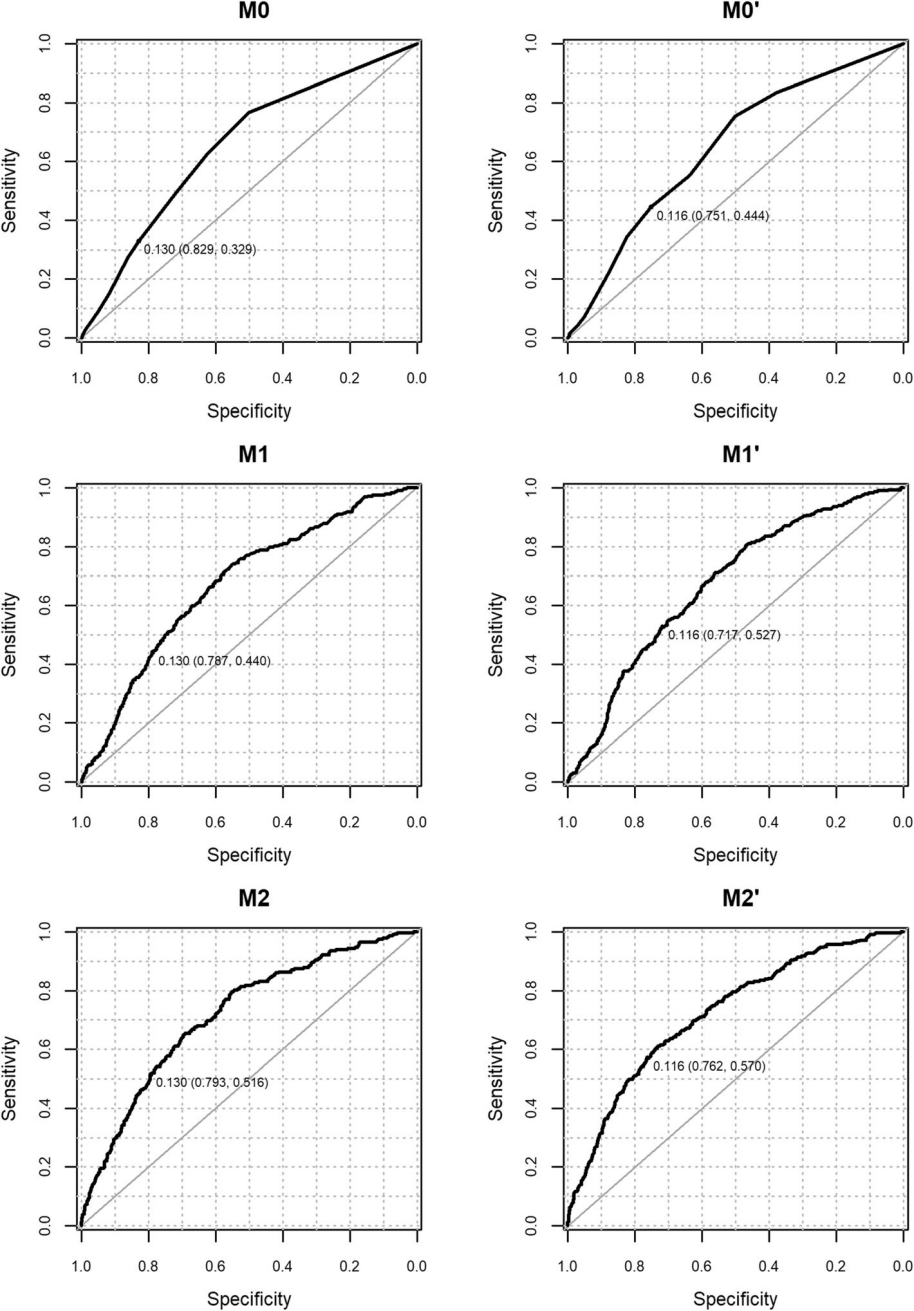
Receiver Operating Characteristic curve for NEWS models (M0,M1,M2) and NEWS2 models (M0’,M1’,M2’) in predicting the risk of COVID-19 following admission in the validation dataset after correcting for calibration-in-the-large. *Note: predicted probability at NEWS or NEWS2 threshold ≥ 5 (sensitivity, specificity) is shown for all models*

We assessed the performance of all models in predicting the risk of COVID-19 in emergency medical admissions (see Table 2 and Fig. 1). The c-statistics for predicting COVID-19 for Model M2’ was the best in class in the development dataset (M0’=0.71; M1’=0.72, M2’: 0.78) (see Table S4 in supplementary material) and the validation dataset (M0’=0.65; M1’=0.67, M2’: 0.72) (see Table 2). The c-statistics for predicting COVID-19 for M0’,M1’,M2’ models was similar to M0,M1,M2 models in the development and validation datasets. Furthermore, all models are shown statistically significant improvement using likelihood ratio tests (see Table S5 in supplementary material).

**Table 2:** Performance of NEWS (M0, M1, M2) and NEWS2 (M0’,M1’,M2’) models for predicting the risk of COVID on admission for validation dataset

Model	Correcting Calibration-in-the-large	Mean Risk Non-COVID	Mean Risk COVID	Scaled Brier Score (%)	c-statistic (95 % CIs)	Calibration-in-the-large (95 % CIs)	Calibration Slope (95 % CIs)
M0	No	0.09	0.11	-14.7	0.65 (0.62 to 0.69)	0.19 (0.06 to 0.32)	0.72 (0.53 to 0.91)
M1	No	0.09	0.12	-12.4	0.67 (0.64 to 0.7)	0.18 (0.05 to 0.31)	0.81 (0.63 to 0.99)
M2	No	0.09	0.16	-8.2	0.72 (0.69 to 0.75)	0.19 (0.06 to 0.32)	0.78 (0.65 to 0.91)
M0	Yes	0.11	0.14	0.5	0.65 (0.62 to 0.69)	-	0.72 (0.53 to 0.91)
M1	Yes	0.11	0.14	1.9	0.67 (0.64 to 0.7)	-	0.81 (0.63 to 0.99)
M2	Yes	0.10	0.18	5.2	0.72 (0.69 to 0.75)	-	0.78 (0.65 to 0.91)
M0`	No	0.09	0.12	-14.2	0.65 (0.61 to 0.68)	0.18 (0.06 to 0.31)	0.69 (0.51 to 0.87)
M1`	No	0.09	0.12	-12.2	0.67 (0.64 to 0.7)	0.17 (0.05 to 0.30)	0.78 (0.60 to 0.96)
M2`	No	0.09	0.17	-6.9	0.72 (0.69 to 0.75)	0.18 (0.05 to 0.31)	0.76 (0.64 to 0.89)
M0`	Yes	0.11	0.14	0.2	0.65 (0.61 to 0.68)	-	0.69 (0.51 to 0.87)
M1`	Yes	0.11	0.14	1.3	0.67 (0.64 to 0.7)	-	0.78 (0.60 to 0.96)
M2`	Yes	0.10	0.19	5.5	0.72 (0.69 to 0.75)	-	0.76 (0.64 to 0.89)

*ARD* absolute risk difference, *CIs* confidence intervals

Table 3 includes the sensitivity, specificity, positive and negative predictive values for all models for predicting COVID-19 for validation dataset. NEWS2 models (M0’,M1’,M2’) had the highest sensitivity but lower specificity compared to NEWS models (M0,M1,M2) because the predicted probability at NEWS2 ≥ 5 (0.116) is lower than at NEWS ≥ 5 (0.13) in the development dataset. Likewise, the performance for development dataset is shown in Table S6.

**Table 3:** Sensitivity analysis of NEWS (M0, M1, M2) and NEWS2 models (M0’, M1’, M2’) for predicting the risk of COVID at threshold NEWS/NEWS2 ≥ 5 (predicted probability of model M0 = 0.130 and M0’ = 0.116 using development dataset) for validation dataset after correcting the calibration-in-the-large

Model	Number of positive cases identified by model	Sensitivity%	Specificity%	PPV	NPV	LR+	LR-
M0	474	32.9 (27.4 to 38.7)	82.9 (81.3 to 84.5)	19.2 (15.7 to 23)	90.9 (89.6 to 92.1)	1.9 (1.6 to 2.3)	0.8 (0.7 to 0.9)
M1	600	44 (38.1 to 50.1)	78.7 (76.9 to 80.4)	20.3 (17.2 to 23.8)	91.9 (90.6 to 93.1)	2.1 (1.8 to 2.4)	0.7 (0.6 to 0.8)
M2	607	51.6 (45.6 to 57.6)	79.3 (77.6 to 81)	23.6 (20.2 to 27.1)	93 (91.8 to 94.1)	2.5 (2.2 to 2.9)	0.6 (0.5 to 0.7)
M0`	681	44.4 (38.5 to 50.5)	75.1 (73.3 to 76.9)	18.1 (15.2 to 21.2)	91.6 (90.3 to 92.9)	1.8 (1.5 to 2.1)	0.7 (0.7 to 0.8)
M1`	781	52.7 (46.6 to 58.7)	71.7 (69.8 to 73.5)	18.7 (16 to 21.6)	92.5 (91.1 to 93.7)	1.9 (1.6 to 2.1)	0.7 (0.6 to 0.7)
M2`	692	57 (51 to 62.9)	76.2 (74.4 to 77.9)	22.8 (19.8 to 26.1)	93.5 (92.3 to 94.6)	2.4 (2.1 to 2.7)	0.6 (0.5 to 0.6)

*PPV *Positive Predictive Value, *NPV *Negative Predictive Value, *LR+ *Positive Likelihood Ratio, *LR- *Negative Likelihood Ratio

Internal validation of these models is shown in Supplemental Digital Content - Figure S6.

The calibration slope was similar and less than one across all NEWS2 (M0’,M1’,M2’) and NEWS (M0,M1,M2) models, which shows overfitting (see Table 2; Fig. 1; Supplemental Digital Content - Table S4 & Figure S7).

However, model M2’ had the highest sensitivity (M2’: 57 % (95 %CI 51-63 %) vs. M0’ 44 % (95 %CI 38-50 %) and M1’ 53 % (95 %CI 47-59 %)) and the highest specificity (M2’:76 % (95 %CI 74-78 %) vs. M0’:75 % (95 %CI 73-77 %) and M1’:72 % (95 %CI 70-74 %)) for the validation dataset at NEWS2 ≥ 5 (see Table 3).

Likewise, model M2 had the highest sensitivity (M2:52 % (95 %CI 46-58 %) vs. M0:33 % (95 %CI 27-38 %) and M1:44 % (95 %CI 38-50 %)) but lowest specificity (M2: 79 % (95 %CI 77-81 %) vs. M0:83 % (95 %CI 81-85 %) and M1:79 % (95 %CI 77-80 %)) for the validation dataset at NEWS2 ≥ 5 (see Table 3).

**Fig. 2:**
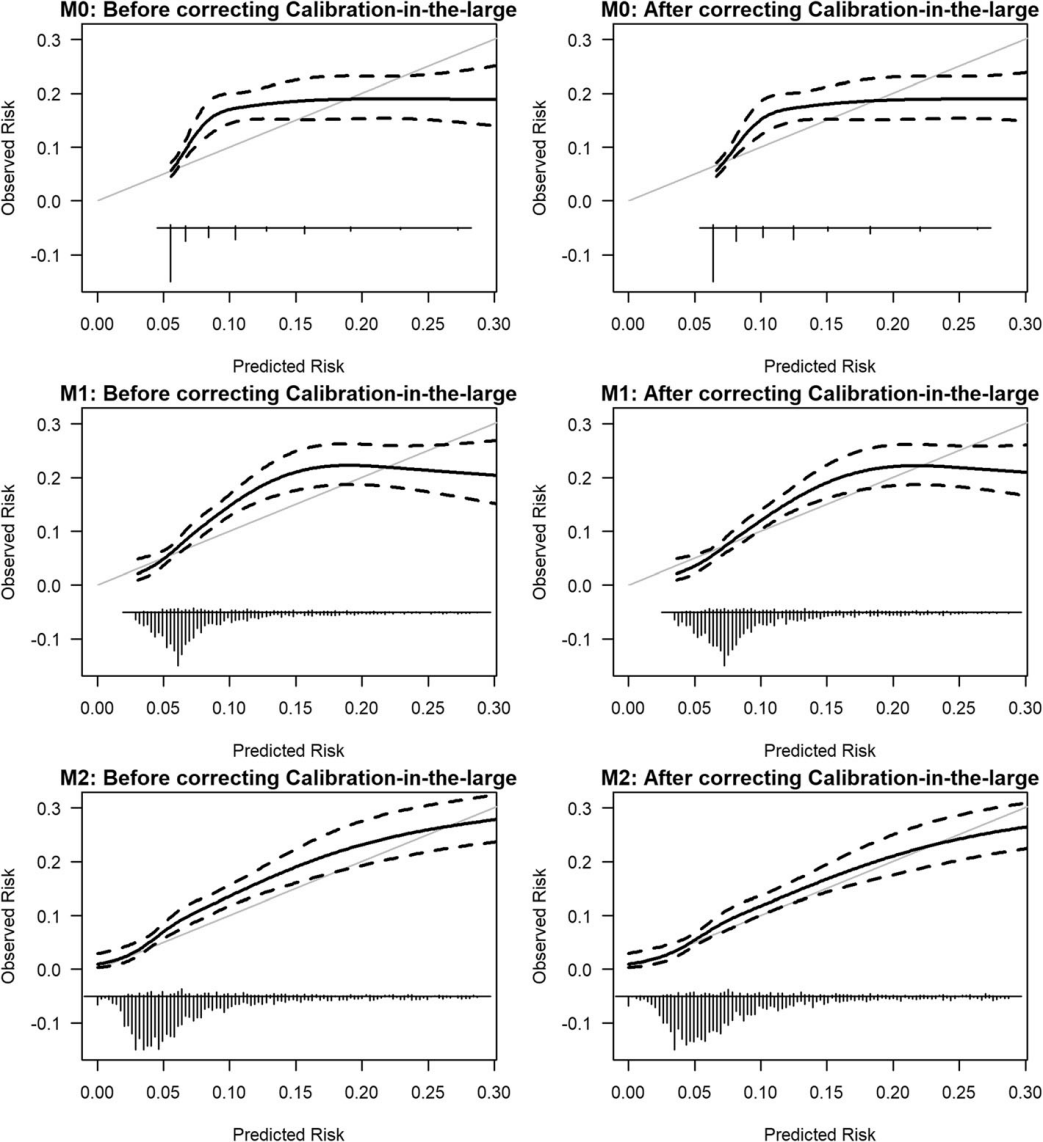
External validation of NEWS models (M0, M1, M2) for predicting the risk of COVID-19. NB: We limit the risk of COVID-19 to 0.30 for visualisation purposes because beyond this point, we have few patients. *The grey solid line shows ideal calibration. The black solid line shows the observed calibration along with 95 % confidence intervals in black dashed lines*

**Fig. 3:**
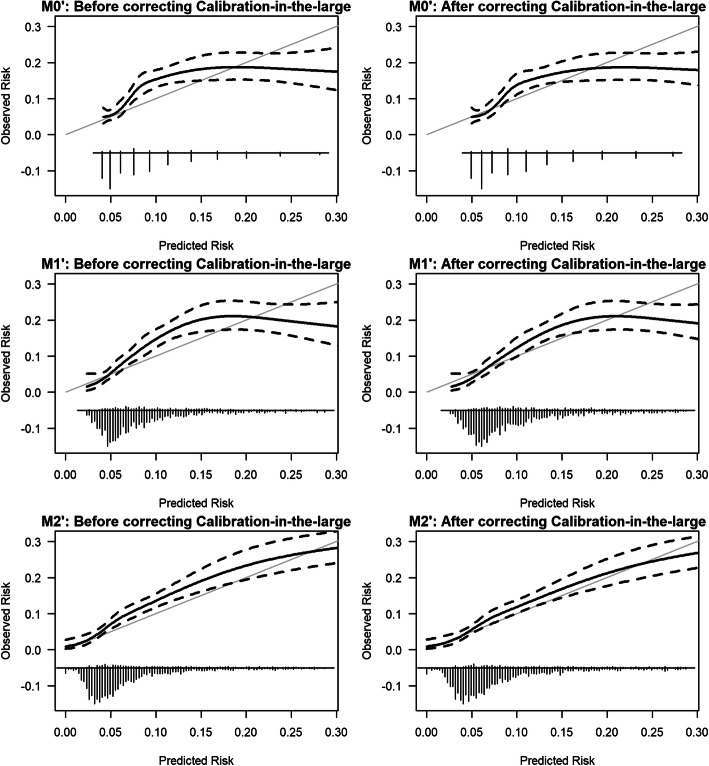
External validation of NEWS2 models (M0’, M1’, M2’) for predicting the risk of COVID-19. NB: We limit the risk of COVID-19 to 0.30 for visualisation purposes because beyond this point, we have few patients. *The grey solid line shows ideal calibration. The black solid line shows the observed calibration along with 95 % confidence intervals in black dashed lines*

Figures 2 and 3 show model calibration improved across the models and that models M2’ and M2 are well-calibrated after correcting for the baseline difference.

**Fig. 4:**
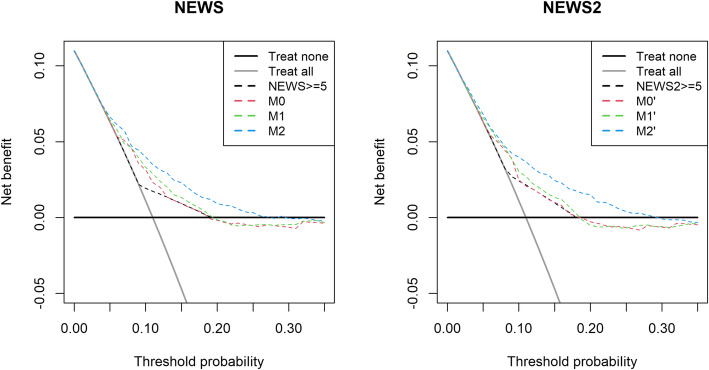
Net Benefit for NEWS models (M0, M1, M2) and NEWS2 models (M0’,M1’,M2’) in predicting the risk of COVID-19 in the validation dataset

Figure 4 shows that model M2’/M2 had the highest net benefit (M2’/M2:0.04 vs. M1’/M1:0.03 and M0’/M0:0.02). As the unit of net benefit is true positives, the model M2’/M2 identified 4 out of 100 COVID-19 admissions, compared to M1’/M1 (3 out of 100) and M0’/M0 (2 out of 100) (see Supplemental Digital Content - Figure S8 for development dataset).

Nevertheless, NEWS/NEWS2 ≥ 5 is the worst performing choice compared to all models (NEWS2: M0’,M1’,M2’ & NEWS: M0,M1,M2).

## Discussion

In this study, we investigated the performance of multiple enhanced NEWS2 models in terms of discrimination and calibration in predicting the risk of COVID-19. Model M0’ uses NEWS2 alone; Model M1’ extends M0’ with age and sex and Model M2’ extends M1’ with all the subcomponents of NEWS2 plus diastolic blood pressure. Equivalent models (M0, M1, M2) using NEWS were also developed but model M2 excluded two parameters that are in NEWS2 but not in NEWS - oxygen flow rate and scale 2 (yes/no).

NEWS2 models (M0’, M1’, M2’) were more sensitive but less specific than NEWS models (M0, M1, M2). Models M2 and M2’ were the best in class, with the highest c-statistics (0.77 and 0.72 respectively). The high negative predictive value suggests models M2 and M2’ may be particularly useful in ruling out COVID-19 early in the patients unplanned admission which is clinically useful because testing for COVID-19 using viral nucleic acid testing is more time consuming than measuring and recording the patients vital signs data sets as defined by NEWS/NEWS2.

A recent systematic review identified five models to detect COVID-19 infection in symptomatic individuals with c-statistics that ranged from 0.87 to 1 [21]. However, despite these high c-statistics, the review authors cautioned against the use of these models in clinical practice because of the high risk of bias and poor reporting of studies which are likely to have led to optimistic results [21]. For example, the majority of studies are with smaller sample size; the lack of external validation and calibration was rarely assessed [21]. Our study addresses these shortcomings. While most of the studies reported an insufficient sample size [21], our study was sufficiently large for developing and validating the models in predicting the risk of COVID-19 [18]. The models were developed using data from one and validated using data from another hospital. We rigorously assessed the internal calibration using bootstrapping approach [22]. Furthermore, calibration slope and calibration-in-the-large are assessed and corrected.

The main advantages of our NEWS/NEWS2 models are that they are designed to incorporate data that are already available in the patient’s electronic health record and so place no additional data collection or computational burden on clinicians and can also be readily automated. Nonetheless, we emphasize that our NEWS/NEWS2 models are not designed to replace clinical judgement. They are intended and designed to support, not subvert, the clinical decision-making process and can be always overridden by clinical concern [5, 23]. The working hypothesis for our models is that their use may enhance situational awareness of COVID-19 by processing information already available without impeding the workflow of clinical staff, especially as our approach offers a faster and less expensive assessment of COVID-19 risk than current laboratory tests. This may be more practical to use in low resource settings or where large numbers of people are to be assessed.

There are limitations in relation to our study. We identified COVID-19 based on ICD-10 code ‘U071’ which was determined by clinical judgment and/or swab test results and so our findings are constrained by the accuracy of these methods [24, 25]. Moreover, we do not have the timing of diagnosis in our data and so we are unable to determine if patients arrived with COVID-19. Our two hospitals are part of the same NHS Trust and this may undermine the generalisability of our findings, and so further external validation may be worthwhile. Another issue related to generalisability is to determine the extent to which mass vaccinations for COVID-19 impact on the accuracy of our models. Finally, an important next phase of this work is to field-test our models by carefully engineering them into routine clinical practice [26, 27] to see if they do support the earlier detection and care of COVID-19 in emergency medical patients without unintended adverse consequences.

## Conclusions

NEWS model M2 and NEWS2 model M2’ appear to provide reasonably accurate predictions of the risk of COVID-19 using routinely collected on-admission NEWS/NEWS2 datasets. The extent to which these models are clinically useful as an early warning system for COVID-19 at the time of admission should be studied.

## Supplementary Information

**Additional file 1: Table S1.** NEWS scoring chart. **Table S2.** NEWS2 scoring chart. **Table S3.** Number of emergency medical admissions included/excluded. **Figure S1.** Escalation policy of deteriorating patients in York Teaching Hospital NHS Foundation Trust. **Figure S2.** Boxplot for continuous covariates without outliers to COVID-19 (Yes/No) for development dataset. **Figure S3.** Scatter plots showing the observed risk of COVID-19 with continuous covariates for the development dataset. **Figure S4.** Boxplot for continuous covariates without outliers to COVID-19 (Yes/No) for validation dataset. **Figure S5.** Scatter plots showing the observed risk of COVID-19 with continuous covariates for validation dataset. **Figure S6.** Internal calibration of NEWS models (M0, M1, M2) and NEWS2 models (M0’,M1’,M2’) for predicting the risk of COVID-19 in the development dataset. **Table S4.** Performance of NEWS models (M0, M1, M2) and NEWS2 models (M0’,M1’,M2’) for predicting the risk of COVID on admission for development dataset. **Table S5.** Likelihood ratio tests for comparing NEWS models (M0, M1, M2) and NEWS2 models (M0’,M1’,M2’) for predicting the risk of COVID on admission for development dataset. **Figure S7.** Receiver Operating Characteristic curve for NEWS models (M0, M1, M2) and NEWS2 models (M0’,M1’,M2’) in predicting the risk of COVID-19 in the development dataset. **Table S6.** Sensitivity analysis of NEWS models (M0, M1, M2) and NEWS2 models (M0’, M1’, M2’) for predicting the risk of COVID at threshold ≥5 of NEWS (predicted probability of model M0 = 0.130) and NEWS2 (predicted probability of model M0’ = 0.116) for development dataset. **Figure S8.** Net Benefit for NEWS models (M0, M1, M2) and NEWS2 models (M0’,M1’,M2’) in predicting the risk of COVID-19 in the development dataset.

## Data Availability

The data that support the findings of this study are available from NHS York hospital trust but restrictions apply to the availability of these data, which were used under license for the current study, and so are not publicly available. However, if anyone is interested in the data, then they should contact the R&D offices in the first instance https://www.research.yorkhospitals.nhs.uk/about-us1/our-directorates/.

## References

[1] Gorbalenya AE, Baker SC, Baric RS, de Groot RJ, Drosten C, Gulyaeva AA (2020). The species Severe acute respiratory syndrome-related coronavirus: classifying 2019-nCoV and naming it SARS-CoV-2. Nat Microbiol.

[2] Onder G, Rezza G, Brusaferro S (2020). Case-fatality rate and characteristics of patients dying in relation to COVID-19 in Italy. JAMA.

[3] Vincent JL, Taccone FS (2020). Understanding pathways to death in patients with COVID-19. Lancet Respir Med.

[4] Weekly statistics for NHS Test and Trace (England) and coronavirus testing (UK): 24 September to 30 September - GOV.UK. https://www.gov.uk/government/publications/nhs-test-and-trace-england-and-coronavirus-testing-uk-statistics-24-september-to-30-september-2020/weekly-statistics-for-nhs-test-and-trace-england-and-coronavirus-testing-uk-24-september-to-30-september. Accessed 14 Oct 2020.

[5] Royal College of Physicians. National Early Warning Score (NEWS): Standardising the assessment of acuteillness severity in the NHS - Report of a working party. 2012.

[6] NHS. Royal College of Physicians: NHS England approves use of National Early Warning Score (NEWS) 2 to improve detection of acutely ill patients. 2017. https://www.rcplondon.ac.uk/news/nhs-england-approves-use-national-early-warning-score-news-2-improve-detection-acutely-ill.

[7] Steyerberg EW. Clinical Prediction Models: A Practical Approach to Development, Validation, and Updating (Statistics for Biology and Health). 2nd Edition. Cham: Springer Nature Switzerland AG; 2020.

[8] Hanley JA, McNeil BJ (1982). The meaning and use of the area under a receiver operating characteristic (ROC) curve. Radiology.

[9] Robin X, Turck N, Hainard A, Tiberti N, Lisacek F, Sanchez JJ-CC (2011). pROC: an open-source package for R and S + to analyze and compare ROC curves. BMC Bioinformatics.

[10] R Development Core Team. R: A language and environment for statistical computing. R Foundation for Statistical Computing. 2015. http://www.r-project.org/.

[11] Steyerberg EW, Harrell FE, Borsboom GJJ, Eijkemans MJ, Vergouwe Y, Habbema JDF (2001). Internal validation of predictive models: Efficiency of some procedures for logistic regression analysis. J Clin Epidemiol.

[12] Harrell FE. rms: Regression Modeling Strategies. 2015. http://cran.r-project.org/package=rms.

[13] National Institute for Health and Care Excellence. Sepsis Quality Standard [QS161]. Published September 2017. https://www.nice.org.uk/guidance/qs161/resources/sepsis-pdf-75545595402181.

[14] NHS England. Sepsis Guidance implementation advice for adults: NHS England, Sept 17. https://www.england.nhs.uk/wp-content/uploads/2017/09/sepsis-guidance-implementation-advice-for-adults.pdf.

[15] Sing T, Sander O, Beerenwinkel N, Lengauer T (2005). ROCR: visualizing classifier performance in R. Bioinformatics.

[16] Vickers AJ, Elkin EB (2006). Decision curve analysis: a novel method for evaluating prediction models. Med Decis Making.

[17] CRAN - Package pmsampsize. https://cran.r-project.org/web/packages/pmsampsize/index.html. Accessed 3 Aug 2020.

[18] Riley RD, Ensor J, Snell KIE, Harrell FE, Martin GP, Reitsma JB, et al. Calculating the sample size required for developing a clinical prediction model. BMJ. 2020;368:m441. 10.1136/bmj.m441. 10.1136/bmj.m441 32188600

[19] Moons KGM, Altman DG, Reitsma JB, Ioannidis JPA, Macaskill P, Steyerberg EW (2015). Transparent Reporting of a multivariable prediction model for Individual Prognosis Or Diagnosis (TRIPOD): Explanation and Elaboration. Ann Intern Med.

[20] StatCorp, Stata (2016). Release 14. Statistical Software.

[21] Wynants L, Van Calster B, Collins GS, Riley RD, Heinze G, Schuit E (2020). Prediction models for diagnosis and prognosis of covid-19: Systematic review and critical appraisal. BMJ.

[22] Harrell FE (2001). Regression modeling strategies: with applications to linear models, logistic regression, and survival analysis.

[23] Balamuth F, Alpern ER, Abbadessa MK, Hayes K, Schast A, Lavelle J (2017). Improving recognition of pediatric severe sepsis in the emergency department: contributions of a vital sign-based electronic alert and bedside clinician identification. Ann Emerg Med.

[24] Corfield AR, Lees F, Zealley I, Houston G, Dickie S, Ward K, et al. Utility of a single early warning score in patients with sepsis in the emergency department. 2012. 10.1136/emermed-2012-202186 23475607

[25] Churpek MM, Snyder A, Han X, Sokol S, Pettit N, Howell MD (2017). Quick Sepsis-related Organ Failure Assessment, Systemic Inflammatory Response Syndrome, and Early Warning Scores for Detecting Clinical Deterioration in Infected Patients outside the Intensive Care Unit. Am J Respir Crit Care Med.

[26] Escobar GJ, Dellinger RP (2016). Early detection, prevention, and mitigation of critical illness outside intensive care settings. J Hosp Med.

[27] Escobar GJ, Turk BJ, Ragins A, Ha J, Hoberman B, LeVine SM (2016). Piloting electronic medical record-based early detection of inpatient deterioration in community hospitals. J Hosp Med.

