# Supplementary material for: Predictive accuracy of enhanced versions of the on-admission National Early Warning Score in estimating the risk of COVID-19 for unplanned admission to hospital: a retrospective development and validation study
Source: BMC Health Serv Res. 2021 Sep 13;21:957. doi: 10.1186/s12913-021-06951-x (PMC8435351; doi:10.1186/s12913-021-06951-x)
Supplement: Supplementary file 1 — Additional file 1: Table S1. NEWS scoring chart. Table S2. NEWS2 scoring chart. Table S3. Number of emergency medical admissions included/excluded. Figure S1. Escalation policy of deteriorating patients in York Teaching Hospital NHS Foundation Trust. Figure S2. Boxplot for continuous covariates without outliers to COVID-19 (Yes/No) for development dataset. Figure S3. Scatter plots showing the observed risk of COVID-19 with continuous covariates for the development dataset. Figure S4. Boxplot for continuous covariates without outliers to COVID-19 (Yes/No) for validation dataset. Figure S5. Scatter plots showing the observed risk of COVID-19 with continuous covariates for validation dataset. Figure S6. Internal calibration of NEWS models (M0, M1, M2) and NEWS2 models (M0’,M1’,M2’) for predicting the risk of COVID-19 in the development dataset. Table S4. Performance of NEWS models (M0, M1, M2) and NEWS2 models (M0’,M1’,M2’) for predicting the risk of COVID on admission for development dataset. Table S5. Likelihood ratio tests for comparing NEWS models (M0, M1, M2) and NEWS2 models (M0’,M1’,M2’) for predicting the risk of COVID on admission for development dataset. Figure S7. Receiver Operating Characteristic curve for NEWS models (M0, M1, M2) and NEWS2 models (M0’,M1’,M2’) in predicting the risk of COVID-19 in the development dataset. Table S6. Sensitivity analysis of NEWS models (M0, M1, M2) and NEWS2 models (M0’, M1’, M2’) for predicting the risk of COVID at threshold ≥5 of NEWS (predicted probability of model M0 = 0.130) and NEWS2 (predicted probability of model M0’ = 0.116) for development dataset. Figure S8. Net Benefit for NEWS models (M0, M1, M2) and NEWS2 models (M0’,M1’,M2’) in predicting the risk of COVID-19 in the development dataset. [file 12913_2021_6951_MOESM1_ESM.pdf]

Supplementary Digital Content

NEWS models (M0, M1, M2)

Model M0

$$Logit(COVID) = -3.064 + 0.232 * NEWS$$

Model M1

$$Logit(COVID) = -3.881 + 0.168 * Male + 0.011 * Age + 0.224 * NEWS$$

Model M2

$$\begin{aligned} Logit(COVID) &= -5.500 + 0.126 * Male + 0.009 * Age - 0.069 * NEWS + 1.770 \\ &* log(Respiratory\ Rate) + 0.286 * Temperature - 0.941 \\ &* log(Systoloic\ pressure) - 0.211 * log(Diastolic\ pressure) + 0.300 \\ &* log(Pulse\ rate) - 0.097 * Oxygen\ Saturations + 1.184 \\ &* Oxygen\ Supplementation - 1.462 * Pain + 0.505 * Voice - 8.393 \\ &* Unconscious \end{aligned}$$

We accounted for baseline difference in risk of COVID-19 in the external validation data by adding (M0: 0.19, M1:0.18, M2:0.19) to the NEWS based models using an iterative procedure described elsewhere[1]

1. **Faisal M, Howes R, Steyerberg EW, Richardson D, Mohammed MA. Using routine blood test results to predict the risk of death for emergency medical admissions to hospital: an external model validation study. QJM [Internet]. 2017 Jan 1 [cited 2017 Oct 2];110(1):27–31. Available from: https://academic.oup.com/qjmed/article-lookup/doi/10.1093/qjmed/hcw110**

NEWS2 models (M0’, M1’, M2’)

Model M0’

$$Logit(COVID) = -3.131 + 0.219 * NEWS2$$

Model M1’

$$Logit(COVID) = -3.946 + 0.181 * Male + 0.011 * Age + 0.213 * NEWS2$$

Model M2’

$$\begin{aligned} Logit(COVID) \\ &= -4.147 + 0.114 * Male + 0.009 * Age + 0.006 * NEWS2 + 1.416 \\ &* log(Respiratory\ Rate) + 0.287 * Temperature - 0.756 \\ &* log(Systoloic\ pressure) - 0.348 * log(Diastolic\ pressure) + 0.167 \\ &* log(Pulse\ rate) - 0.099 * Oxygen\ Saturations + 0.797 \\ &* Oxygen\ Supplementation - 2.185 * Pain + 0.120 * Voice - 8.889 \\ &* Unconscious + 0.390 * Baseline\ Confusion + 0.270 * New\ Confusion \\ &- 0.868 * Scale2 + 0.046 * Oxygen\ Flow\ Rate \end{aligned}$$

We accounted for baseline difference in risk of COVID-19 in the external validation data by adding (M0’: 0.18, M1’:0.17, M2’:0.18) to the NEWS2 based models using an iterative procedure described elsewhere[1]

1. **Faisal M, Howes R, Steyerberg EW, Richardson D, Mohammed MA. Using routine blood test results to predict the risk of death for emergency medical admissions to hospital: an external model validation study. QJM [Internet]. 2017 Jan 1 [cited 2017 Oct 2];110(1):27–31. Available from: https://academic.oup.com/qjmed/article-lookup/doi/10.1093/qjmed/hcw110**

Table S1:NEWS scoring chart

Physiological Parameters	3	2	1	0	1	2	3
Respiration Rate	≤8		9 - 11	12 - 20		21 - 24	≥25
Oxygen Saturations	≤91	92 - 93	94 - 95	≥96			
Any Supplemental Oxygen		Yes		No			
Temperature	≤35.0		35.1 - 36.0	36.1 - 38.0	38.1 - 39.0	≥39.1	
Systolic BP	≤90	91 - 100	101 - 110	111 - 219			≥220
Heart Rate	≤40		41 - 50	51-90	91 - 110	111 - 130	≥131
Level of Consciousness				Alert			Voice, Pain, or Unconscious

Table S2: NEWS2 scoring chart

Physiological Parameters	3	2	1	0	1	2	3
Respiration Rate	≤8		9 - 11	12 - 20		21 - 24	≥25
SpO2 Scale 1 (%)	≤91	92 - 93	94 - 95	≥96			
SpO2 Scale 2 (%)	≤83	84 - 85	86 - 87	88 - 92<br>≥93 on Air	93 – 94 on oxygen	95 – 96 on oxygen	≥97 on oxygen
Oxygen Saturations	≤91	92 - 93	94 - 95	≥96			
Air or oxygen?		Oxygen		Air			
Temperature	≤35.0		35.1 - 36.0	36.1 - 38.0	38.1 - 39.0	≥39.1	
Systolic BP	≤90	91 - 100	101 - 110	111 - 219			≥220
Heart Rate	≤40		41 - 50	51-90	91 - 110	111 - 130	≥131
Level of Consciousness				Alert			Voice, Pain, Confusion, or Unconscious

The NEWS [https://www.rcplondon.ac.uk/projects/outputs/national-early-warning-score-news] is based on a scoring system in which a score is allocated to vital signs physiological measurements already undertaken when patients present to or are being monitored in hospital. A score is allocated to each as they are measured, the magnitude of the score reflecting how extreme the parameter varies from the norm. This score is then aggregated, and uplifted for people requiring oxygen.

Characteristic	Development dataset (YH)	Validation dataset (SH)	All
	N (%)	N (%)	N (%)
Total emergency medical discharges between 11 Mar 20 to 13 June 20	3952	2528	6480
Excluded: No NEWS recorded (%)	13 (0.3)	6 (0.2)	19 (0.3)
Excluded: First NEWS after 24 hours of admission (%)	15 (0.4)	2 (0.1)	17 (0.3)
Total excluded (%)	28 (0.7)	8 (0.3)	36 (0.6)
Total included (%)	3924 (99.3)	2520 (99.7)	6444 (99.4)

Table S3 Number of emergency medical admissions included/excluded

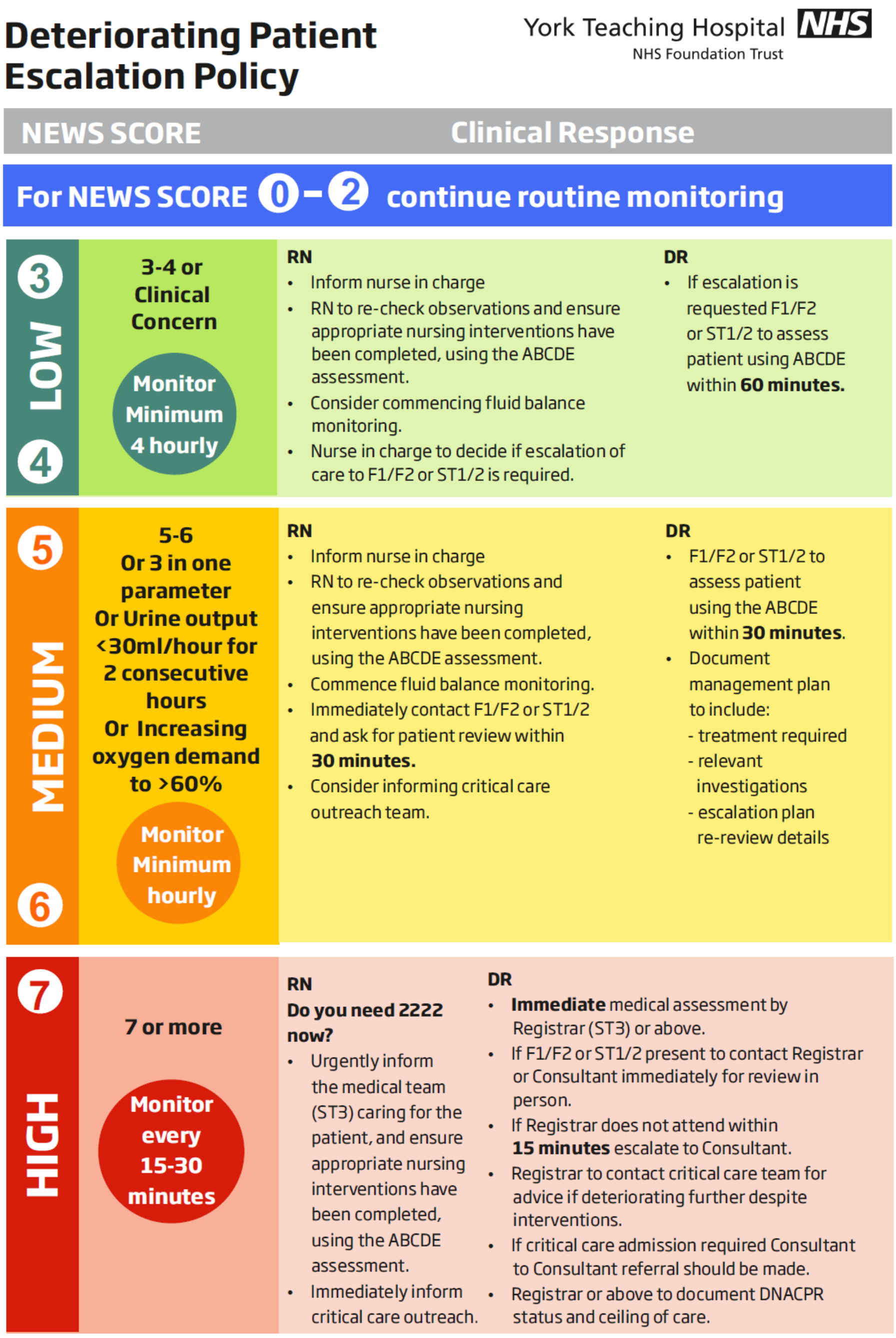

Figure S1 Escalation policy of deteriorating patients in York Teaching Hospital NHS Foundation Trust

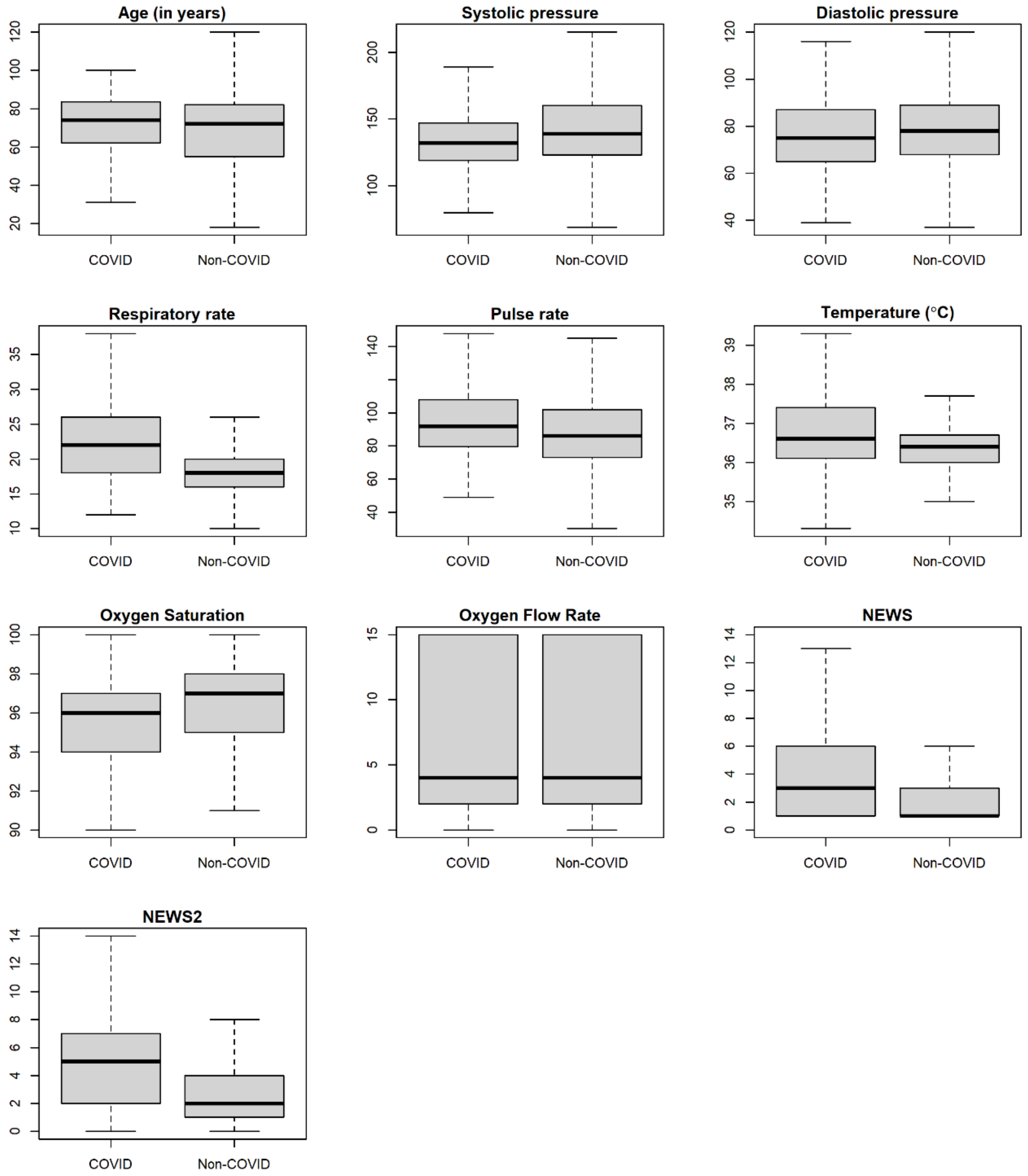

Figure S2 Boxplot for continuous covariates without outliers to COVID-19 (Yes/No) for development dataset.

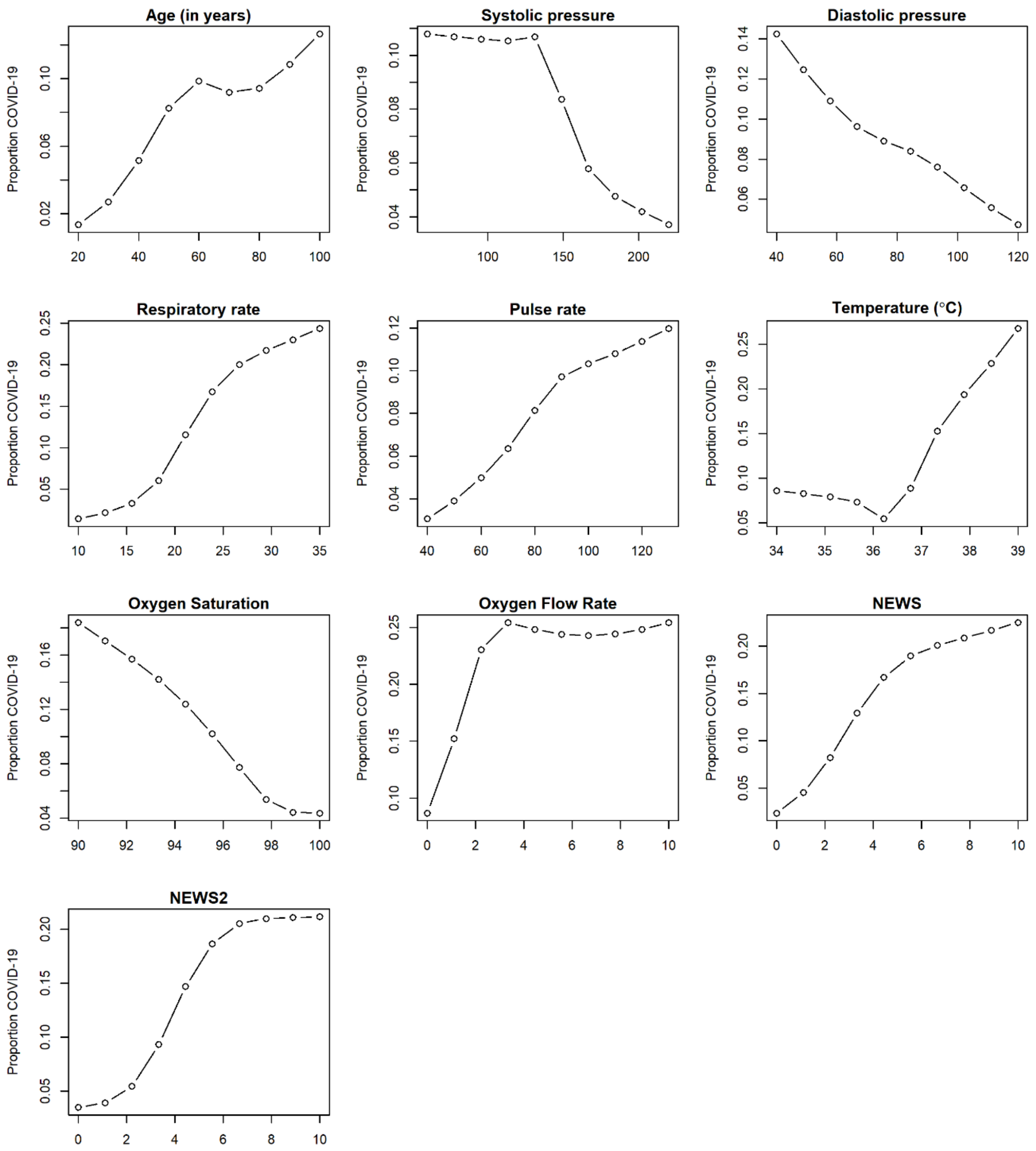

Figure S3 Scatter plots showing the observed risk of COVID-19 with continuous covariates for the development dataset.

NB: y-axis range changes in each plot.

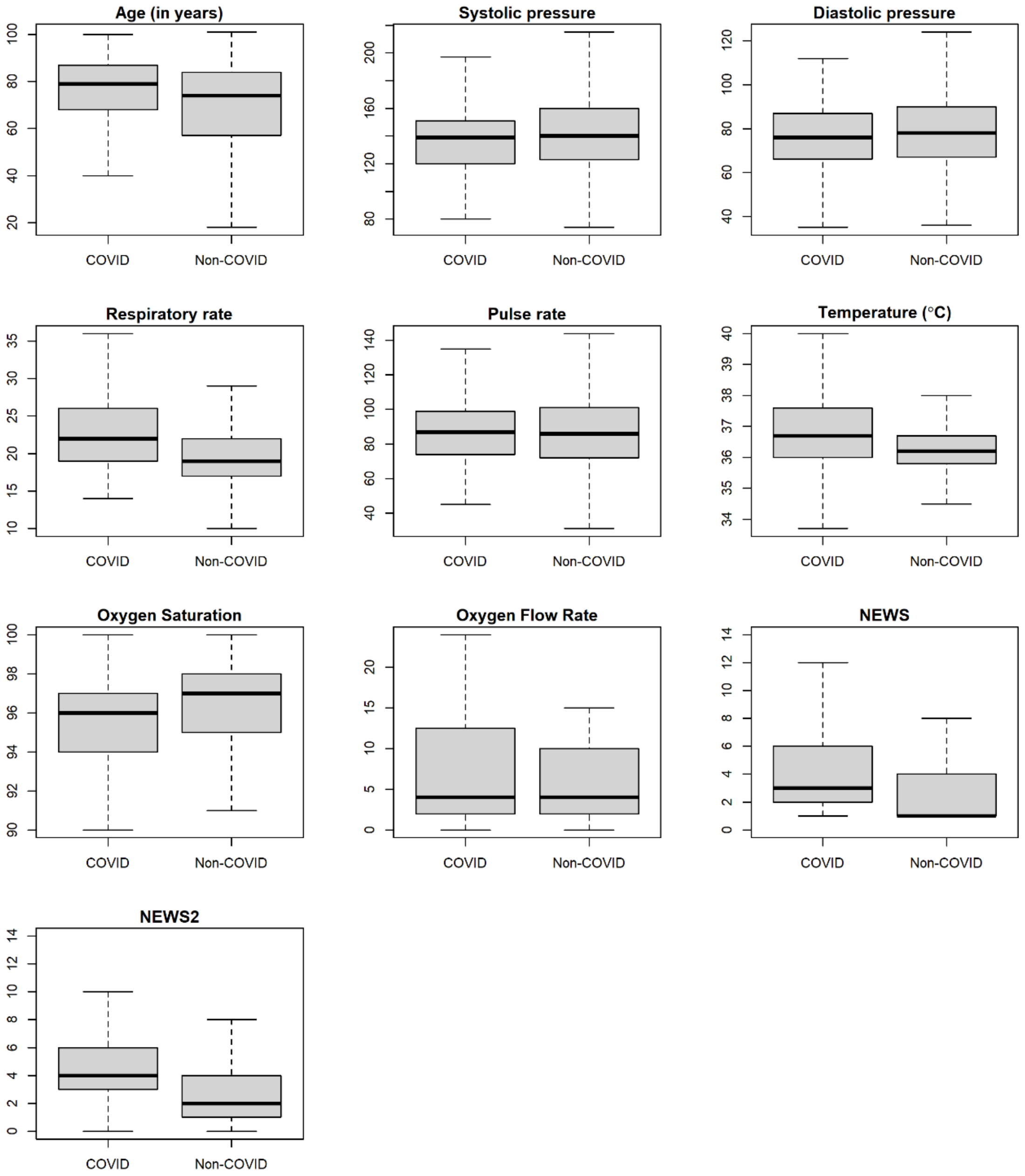

Figure S4 Boxplot for continuous covariates without outliers to COVID-19 (Yes/No) for validation dataset.

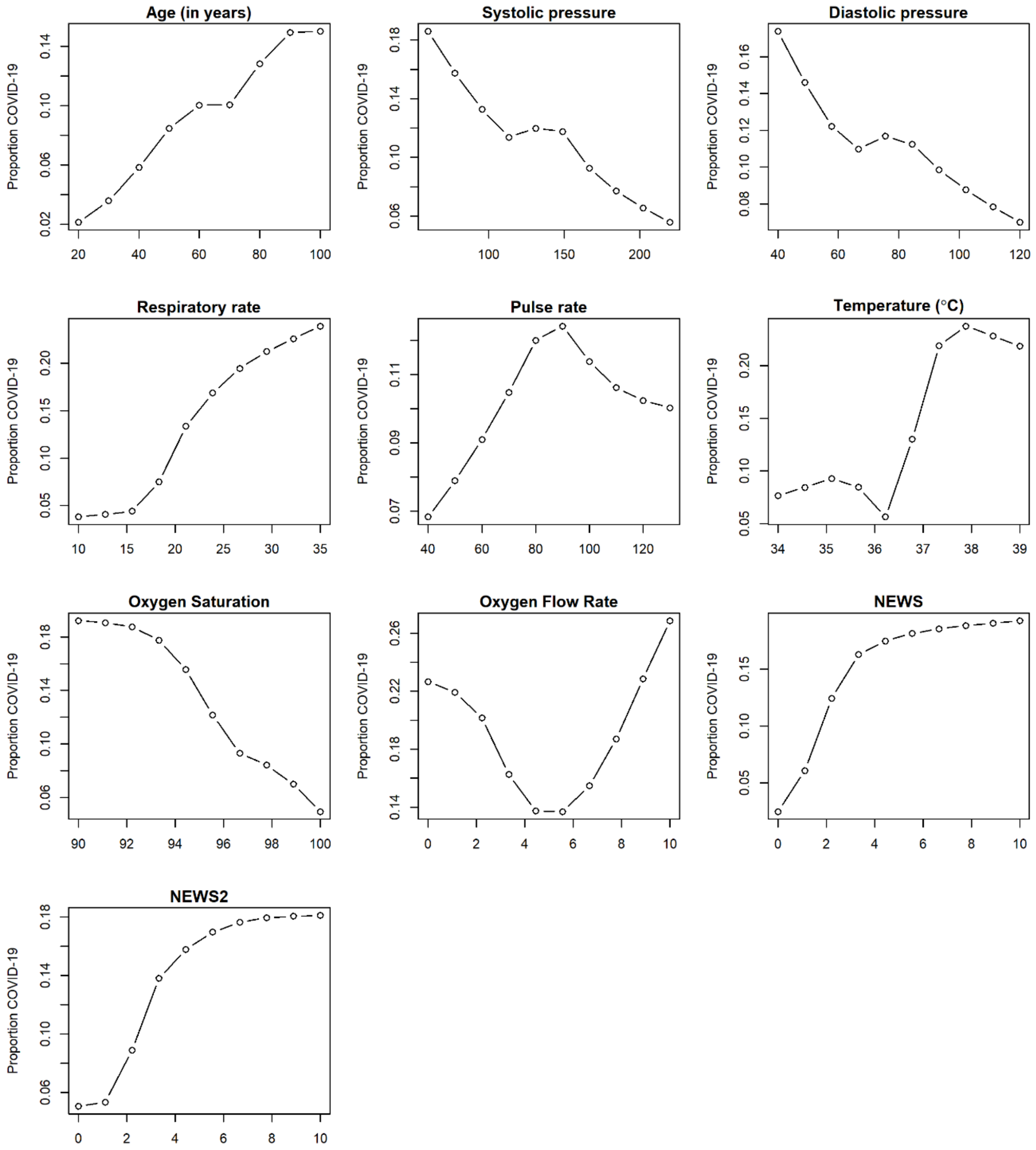

Figure S5 Scatter plots showing the observed risk of COVID-19 with continuous covariates for validation dataset

NB: y-axis range changes in each plot.

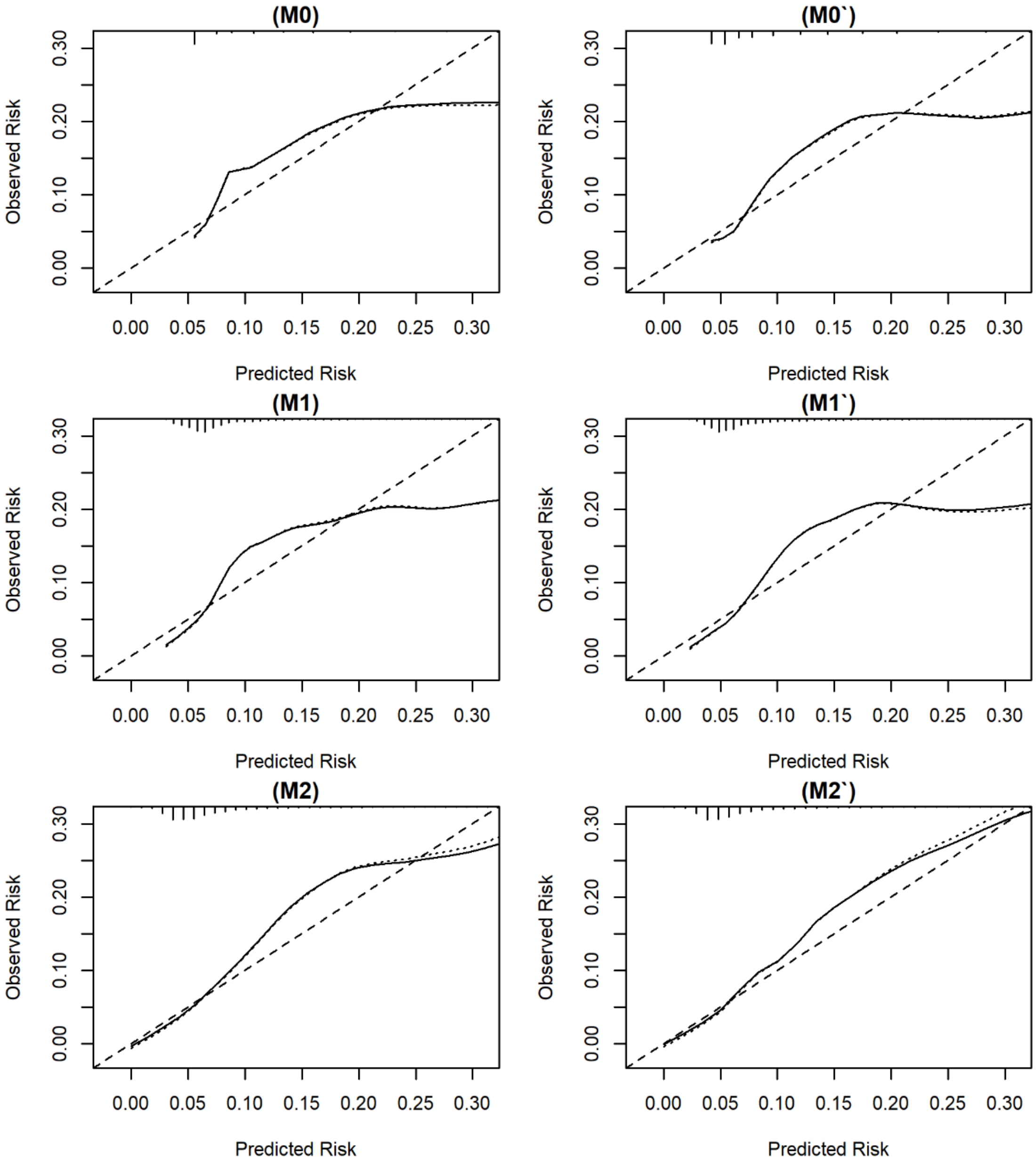

Figure S6 Internal calibration of NEWS models (M0, M1, M2) and NEWS2 models (M0',M1',M2') for predicting the risk of COVID-19 in the development dataset

NB: We limit the risk of COVID-19 to 0.30 for visualisation purposes because beyond this point, we have few patients. The dashed line shows ideal calibration. The dotted line shows apparent calibration and the solid line shows bias-corrected calibration.

Model	Development dataset					
	Mean Risk Non-COVID	Mean Risk COVID	Scaled Brier Score (%)	c-statistic (95% CIs)	Optimism-corrected c-statistic	AIC
**M0**	**0.08**	**0.12**	**3.41**	**0.69 (0.67 to 0.72)**	**0.70**	**2197**
**M1**	**0.08**	**0.13**	**3.56**	**0.71 (0.68 to 0.74)**	**0.71**	**2189**
**M2**	**0.08**	**0.18**	**9.08**	**0.77 (0.75 to 0.8)**	**0.77**	**2049**
**M0`**	**0.08**	**0.13**	**3.89**	**0.71 (0.68 to 0.74)**	**0.71**	**2171**
**M1`**	**0.08**	**0.13**	**3.99**	**0.72 (0.7 to 0.75)**	**0.72**	**2162**
**M2`**	**0.08**	**0.19**	**10.16**	**0.78 (0.75 to 0.81)**	**0.77**	**2034**

Table S4: Performance of NEWS models (M0, M1, M2) and NEWS2 models (M0’,M1’,M2’) for predicting the risk of COVID on admission for development dataset

CIs: confidence intervals

**Comparison**	**Likelihood ratio test**	**Degree of freedom (DF)**	**P-value**
M0 vs M1	12.26	2	0.002
M0 vs M2	171.78	12	<0.001
M1 vs M2	159.52	10	<0.001
M0’ vs M1’	12.31	2	0.002
M0’ vs M2’	168.51	16	<0.001
M1’ vs M2’	156.20	14	<0.001

Table S5: Likelihood ratio tests for comparing NEWS models (M0, M1, M2) and NEWS2 models (M0’,M1’,M2’) for predicting the risk of COVID on admission for development dataset

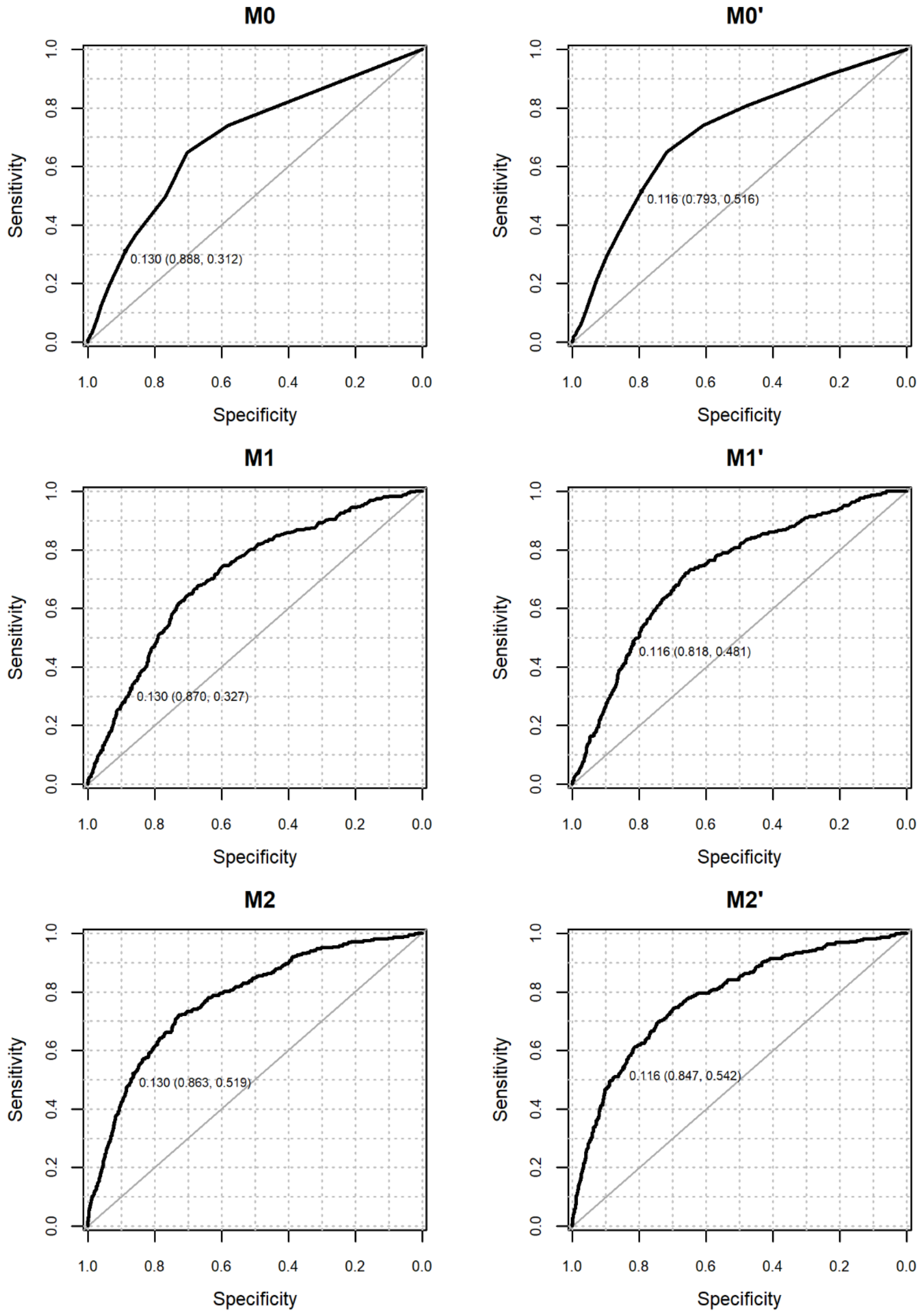

Figure S7 Receiver Operating Characteristic curve for NEWS models (M0, M1, M2) and NEWS2 models (M0',M1',M2') in predicting the risk of COVID-19 in the development dataset

Note: predicted probability at NEWS (or NEWS2) threshold ≥5 (sensitivity, specificity) is shown for all models.

Model	Number of positive cases identified by model	Sensitivity%	Specificity%	PPV	NPV	LR+	LR-
M0	508	31.2<br>(26.3 to 36.4)	88.8<br>(87.7 to 89.8)	21.1<br>(17.6 to 24.9)	93.1<br>(92.2 to 93.9)	2.8<br>(2.3 to 3.3)	0.8<br>(0.7 to 0.8)
M1	577	32.7<br>(27.7 to 37.9)	87<br>(85.9 to 88.1)	19.4<br>(16.3 to 22.9)	93.1<br>(92.2 to 93.9)	2.5<br>(2.1 to 3)	0.8<br>(0.7 to 0.8)
M2	669	51.9<br>(46.5 to 57.3)	86.3<br>(85.1 to 87.4)	26.6<br>(23.3 to 30.1)	94.9<br>(94.1 to 95.7)	3.8<br>(3.3 to 4.3)	0.6<br>(0.5 to 0.6)
M0`	919	51.6<br>(46.2 to 57)	79.3<br>(77.9 to 80.6)	19.3<br>(16.8 to 22)	94.5<br>(93.6 to 95.3)	2.5<br>(2.2 to 2.8)	0.6<br>(0.5 to 0.7)
M1`	818	48.1<br>(42.7 to 53.5)	81.8<br>(80.5 to 83)	20.2<br>(17.5 to 23.1)	94.3<br>(93.4 to 95.1)	2.6<br>(2.3 to 3)	0.6<br>(0.6 to 0.7)
M2`	735	54.2<br>(48.8 to 59.6)	84.7<br>(83.4 to 85.8)	25.3<br>(22.2 to 28.6)	95.1<br>(94.3 to 95.8)	3.5<br>(3.1 to 4)	0.5<br>(0.5 to 0.6)

Table S6 Sensitivity analysis of NEWS models (M0, M1, M2) and NEWS2 models (M0’, M1’, M2’) for predicting the risk of COVID at threshold ≥5 of NEWS (predicted probability of model M0 = 0.130) and NEWS2 (predicted probability of model M0’ = 0.116) for development dataset.

PPV=Positive Predictive Value; NPV= Negative Predictive Value; LR+=Positive Likelihood Ratio; LR-=Negative Likelihood Ratio

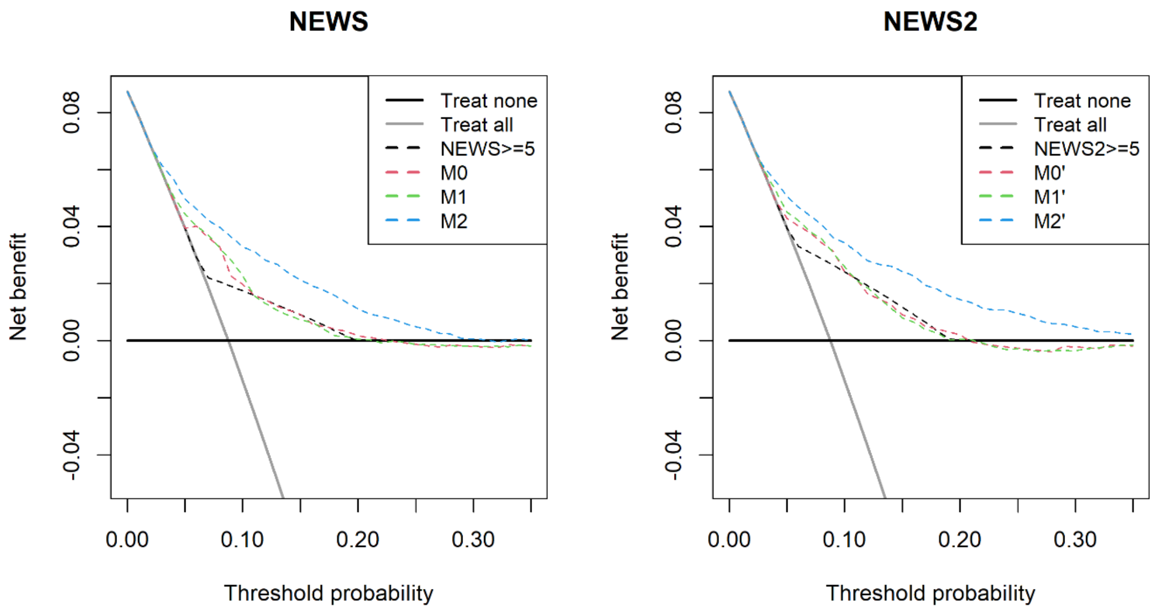

Figure S8 Net Benefit for NEWS models (M0, M1, M2) and NEWS2 models (M0’,M1’,M2’) in predicting the risk of COVID-19 in the development dataset.
